# The Belgian Obstetric Surveillance System to monitor severe maternal morbidity

**Published:** 2017-12

**Authors:** G Vandenberghe, K Roelens, V Van Leeuw, Y Englert, M Hanssens, H Verstraelen

**Affiliations:** Department of Obstetrics & Gynaecology, Ghent University Hospital, 9000 Ghent, Belgium; Department of Obstetrics & Gynaecology, Ghent University Hospital, 9000 Ghent, Belgium; Perinatal Epidemiology Center (Centre d’Épidémiologie Périnatale, CEpiP), 1070 Brussels, Belgium; School of Public Health, Université Libre de Bruxelles (ULB), 1050 Brussels, Belgium; Perinatal Epidemiology Center (Centre d’Épidémiologie Périnatale, CEpiP), 1070 Brussels, Belgium; Faculty of Medicine, Research Laboratory on Human Reproduction, Université Libre de Bruxelles (ULB),1050 Brussels, Belgium; Department of Obstetrics & Gynaecology, Leuven University Hospital, 300 Leuven, Belgium; Department of Obstetrics & Gynaecology, Ghent University Hospital, 9000 Ghent, Belgium

**Keywords:** Severe maternal morbidity, maternal near miss, quality of care, obstetric health care, obstetric surveillance system, population-based

## Abstract

**Background:**

In 2011 the Belgian Obstetric Surveillance System (B.OSS) was set up to monitor severe maternal morbidity in Belgium.

**Aim:**

The aim of B.OSS is to get an accurate picture of the obstetric complications under investigation and secondly, to improve the quality and safety of obstetric care in Belgium by practical recommendations based on the results.

**Methodology:**

Data are obtained through prospective active collection of cases by a monthly call according to the principle of nothing-to-report, along with data collection forms that confirm the diagnosis and gather detailed information. Data-collection occurs web-based since August 2013 through www.b-oss.be.

**Results:**

B.OSS achieves excellent participation rates and response rates. The results of the first registration round are gradually brought out by means of scientific publications and presentations, biennial reports, newsletters and the website. The international comparison of results within the International Network of Obstetric Survey Systems (INOSS) gives important added value. No alternative mandatory data sources are appropriate to check for underreporting.

**Conclusions:**

B.OSS is successful in monitoring severe maternal morbidity thanks to the willingness of the Belgian OB-GYNs. The results of the first studies suggest the need to develop nationally adopted guidelines. Furthermore, the results invite to critically evaluate the current organisation of obstetric health care in Belgium. B.OSS aims to monitor the impact on patient safety in future surveys, when guidelines and recommendations are put into practice.

## Methodology: How does the Belgian Obstetric Surveillance System work?

### Institute

The Belgian Obstetric Surveillance System, briefly B.OSS, is initiated with support of the College of Physicians for Mother and Newborn, a consultative body of the Federal Public Service of Health. B.OSS is endorsed by the two professional associations for OB-GYNs (Vlaamse Vereniging voor Obstetrie en Gynecologie (VVOG) and Groupement des Gynécologues Obstétriciens de Langue Française de Belgique (GGOLFB)), and by the perinatal registries SPE and CEpiP. The College operates as the steering committee of B.OSS. In 2017, a formal scientific board is constituted with representatives of SPE, CEpiP, the Belgian Health Care Knowledge Centre (KCE), the Scientific Institute of Public Health (WIV-ISP) and the College. Daily reporting and data collection tasks are carried out by two cooperating teams: one in Flanders, another in Brussels and Wallonia.

### Data collection

B.OSS has adopted the methodology for case reporting developed by the UK Obstetric Surveillance System (UKOSS)(Knight et al., 2005). An appointed contact person (OB-GYN or senior midwife) in each participating maternity unit is invited by monthly mailing to report a selected number of rare obstetric complications that occurred in the preceding month or alternatively to state that there was ‘nothing to report’, as is mostly the case. In the event of a case being reported, the contact person is asked to complete an extensive data collection form. In case of incomplete reporting, the contact person is encouraged repeatedly by email and phone to provide the missing data.

**Figure 1:**
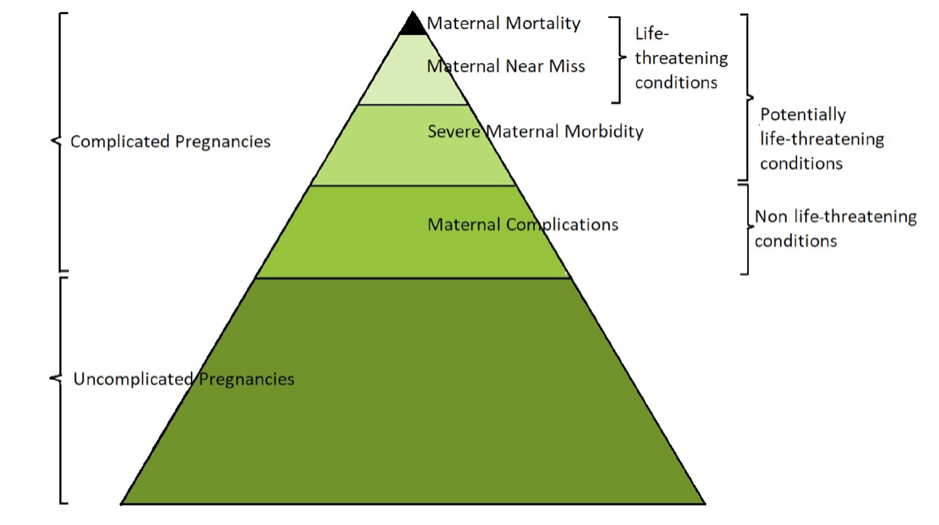
— The continuum from uncomplicated pregnancy to maternal mortality. Adapted from The Pyramid of Disease. PowerPoint Presentation. Introduction to UKOSS. Retrieved from https://www.npeu.ox.ac.uk/ukoss. and The spectrum of morbidity: from noncomplicated pregnancies to maternal death. Say et al., (2009).

### Website www.b-oss.be

Initially, the data of the reported cases were obtained through use of a standardised form, filled out electronically or on hard copy. Web-based data- collection was gradually introduced following the launch of the B.OSS website (www.b-oss.be) in August 2013, facilitating monthly reporting and completion of data collection forms online. Monthly emails calling to report for the previous month are generated automatically, with reminders for missing reporting forms and incomplete data collection forms. Restricted access to the website is provided to the appointed contact person, one per maternity unit, via a personal login. They have access to the reporting forms and data collection forms of their maternity unit. Data protection is secured by the use of anonymous hash codes, replacing person- identifiable information such as the woman’s name, date of birth or hospital number.

### Registered variables

The data collection forms seek information on maternal characteristics, medical, surgical and obstetric history, details of the index pregnancy, details of the delivery, the circumstances of the adverse event, its management and the outcome for mother and neonate. The data are retrieved in retrospect from the woman’s case notes.

### Data analysis

The collected data are coded and exported as comma-separated values (csv) files, then cleaned and processed into operational SPSS data files. Data- analysis is performed using IBM SPSS statistics (IBM Corp. Released 2013. IBM SPSS Statistics for Windows, Version 22.0. Armonk, NY: IBM Corp), EpiTools epidemiological calculators (Sergeant, ESG, 2017. Epitools epidemiological calculators. Ausvet Pty Ltd. Available at: http://epitools.ausvet.com.au) and MedCalc for Windows, version 15.0 Software (MedCalc Software, Ostend, Belgium).

The prevalence of the obstetric events targeted is estimated using as denominator the total number of deliveries in Belgium during the survey period, corrected for the maternity units that did not participate. Reference data are obtained from the perinatal registries (SPE and CEpiP) when available.

### Ethics approval

The B.OSS methodology was approved by the Medical Ethics Committee of Ghent University Hospital (EC UZG 2012/734; B670201215359) and by the Medical Ethics Committee of the Erasme University Hospital, Brussels (EC ULB 2012/111; B406201213660) at the beginning. The Medical Ethics Committee of the Ghent University Hospital became Central Ethics Committee in 2015 (EC UZG 2015/1470; B670201526875). The women eligible for inclusion are informed by their OB- GYN and offered an information letter enabling them to opt-out. Confidentiality is guaranteed for mother, provider and hospital. Person-identifiable information is eliminated from data-analysis.

## Results: What is the output of the Belgian Obstetric Surveillance System?

### Participation rate and response rate

**Figure 2:**
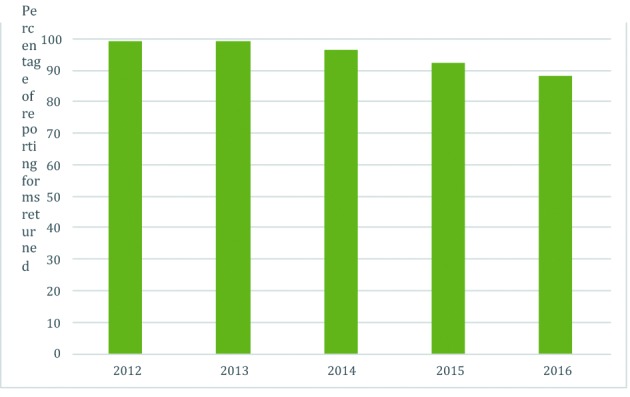
— Yearly response rate of the Belgian Obstetric Surveillance System in percentage.

At start, 110 of 113 Belgian maternity units (97.3 %) formally agreed to participate in B.OSS; the three remaining maternity units have been recruited since. The number of maternity units dropped through the years as a result of merging and closure, with 106 of 107 (99%) participating in January 2017. B.OSS achieved an excellent response rate (the number of monthly reporting forms returned) at start in 2012-2013 (98.9%), which slightly dropped in the following years (Fig. 2).

### Completed and ongoing studies

B.OSS completed a two-year study of uterine rupture (2012-2013) and peripartum hysterectomy and/or arterial embolisation of the uterine arteries (2012-2013) and a three-year study of eclampsia (2012-2014). Currently B.OSS is conducting a four-year study of antenatal pulmonary embolism (APE) (2015-2018) and is participating in the INOSS international studies of spontaneous haemoperitoneum in pregnancy (SHiP) (2015-2017) and anaphylaxis in pregnancy (2016-2018).

### Publications, newsletters, biennial reports and website

The results of the first registration round are brought out gradually. The prevalence, risk factors, management and outcome of the obstetric complications under investigation are communicated towards obstetric and other health care providers by means of scientific publications and presentations. (Vandenberghe et al., 2016; 2017) A newsletter is sent out every six months, providing intermediate results and practical information, besides asking for feedback and ideas for improvement. A biennial report summarising the achievements of B.OSS in the previous two years, is distributed to all OB-GYNs and maternity units. The website (http://www.b-oss.be) provides information on the background and methodology of B.OSS. Study protocols, data collection forms, the patient information letter, scientific publications and presentations of B.OSS- results are freely accessible. The contact persons have restricted access to the newsletters, biennial reports and a continuously updated response rate.

### Validation of data

Despite monthly emails and frequent reminders, underreporting of cases cannot be excluded.

Underreporting is attributed to the non-mandatory participation of busy clinicians and to the case’s nature: severe maternal complications and near miss cases, maybe evoked by some degree of concern on the quality of care.

We consulted two national data sources, collecting medical data on a mandatory basis, that could possibly serve as a control for underreporting for the data recorded by B.OSS. Firstly, the Belgian hospital discharge register (Minimale Ziekenhuis Gegevens (MZG), Résumé Hospitalier Minimum (RHM)), which used the International Classification of Diseases, Ninth Revision, Clinical Modification (ICD- 9-CM) (CDC, 2013) codes up to January 2015. From this data source we derived the total number of peripartum hysterectomy, eclampsia and uterine rupture, indicated by clinicians from January until December 2012. The number of arterial embolisations derived from the hospital discharge register was not accurate, due to the lack of a specific ICD-9-cm code indicating the embolisation of the uterine arteries. As shown in Table I, all numbers from the hospital discharge register exceeded the numbers derived by B.OSS during the same period, even twice (eclampsia) or three times (uterine rupture). This is in line with the findings of true validation studies, such as a study from Denmark (Thisted et al., 2014) in which only 60.4% of the women registered with an ICD-10 code for uterine rupture during labour actually had a uterine rupture. Likewise, a validity study from the United States demonstrated a high positive predictive value (PPV) for ICD-9-cm codes indicating procedures, such as hysterectomy (PPV 100%, 95% CI 86-100%) and a low PPV for ICD-9-cm codes indicating conditions that require the interpretation of clinical information, such as eclampsia (PPV 55%, 33-76%) (Sigakis et al., 2016). We trust that the methodology of the obstetric surveillance system, through monthly reporting and detailed data collection forms, should be more precise than the hospital discharge register.

**Table I:** Number of hospital stays for pregnancy, delivery or abortion, with report of severe obstetric complication in 2012 by the Belgian Hospital Discharge Register; compared to the number registered by the Belgian Obstetric Surveillance System (B.OSS).

Severe obstetric complication	Peripartum hysterectomy* n	Peripartum § embolisation n	Eclampsia$ n	Uterine rupture#
Belgian Hospital Discharge Register (MZG, RHM)	57	24	54	116
B.OSS	44	53	24	41

* ICD-9-CM codes: 68.31,68.39, 68.41, 68.49, 68.51,68.59, 68.61, 68.69, 68.71, 68.79, 68.9

§ ICD-9-CM codes: 38.86, 39.79. The codes 68.24 and 68.25: uterine artery embolization (UAE) with coils and without coils, were not yet available.

$ ICD-9-CM codes : 642.6x

# ICD-9-CM codes : 665.0, 665.1

The second data source theoretically allowing control for underreporting is the Inter Health Insurance Funds Agency (het InterMutualistisch Agentschap (IMA), l’Agence InterMutualiste (AIM))(IMA-AIM, 2017). The IMA-AIM records administrative and billing data of all Belgian inhabitants affiliated to the seven Belgian Health Insurance funds. The database of IMA-AIM includes information of every medical act that was carried out and reimbursed in Belgium based on a specific nomenclature code, such as the timing and location of the medical act and (coded) patient data. From this data source we derived the total number of hysterectomies and arterial embolisations carried out from January 2012 until December 2013 within 4 weeks following a vaginal birth or caesarean section. In Table II is shown that the number of peripartum hysterectomies recorded by IMA-AIM was almost equal, while the number of arterial embolisations largely exceeded the number reported by B.OSS. Possible explanations for this discrepancy are the accuracy of the nomenclature code, which is not specific for embolisation of the uterine arteries, and the hypothesis that in some cases embolisation catheters were placed prophylactically before planned caesarean sections with expected high risk of obstetric haemorrhage while the actual arterial embolisation did not need to be carried out. This data-source could not provide data of uterine ruptures or eclampsia, because those complications do not involve a specific medical act with traceable nomenclature code. Hence, the IMA-AIM database may only be of use to validate surveys of procedures indicative of severe maternal morbidity.

**Table II:** Number of peripartum hysterectomies and peripartum arterial embolisations in 2012-2013 recorded by IMA-AIM; compared to the number registered by the Belgian Obstetric Surveillance System (B.OSS).

Severe obstetric complication	Peripartum hysterectomy	Peripartum embolisation
IMA – AIM*	77	139
B.OSS	79	96

*IMA-AIM records billing data of Belgian inhabitants affiliated to the seven Belgian Health Insurance funds, using nomenclature codes.

### International comparison and benchmarking

The use of similar methods and data collection forms based on the UKOSS, enables the comparison of the Belgian data with the results of studies performed by obstetric surveillance systems in other countries. The international comparison of prevalences, risk factors, management and outcome adds important value on top of the conclusions that can be made based on national data.

Within the INOSS network, we conducted an international comparison study of uterine rupture between nine countries, resulting in the largest prospective population-based report of uterine rupture in high-income countries (unpublished data, paper submitted October 2017). This study reports a prevalence of uterine rupture in women with previous caesarean section of 22 (95% CI 2124) per 10,000 deliveries, ranging from 8 (95% CI 6-11) to 68 (95% CI 56-83) per 10,000 deliveries. This wide range in prevalence between countries appears to be, amongst others, a consequence of the range in trial of labour after caesarean section (TOLAC) rates between countries (from 47% to 72%). Benchmarking population-based TOLAC rates opposed to uterine rupture rates reveals that countries with high TOLAC rates pay a slightly higher price in uterine ruptures per TOLAC. This international comparison study suggests a need to critically appraise TOLAC policies in each country: first, the selection of appropriate candidates and second, the management of labour in women who decided to undergo TOLAC. With regard to Belgium, the prevalence of uterine rupture in women with previous caesarean section (21 (95% CI 16-27) per 10,000 deliveries) is in the lower-middle of prevalences, while the TOLAC rate (percentage of women with previous caesarean section who underwent TOLAC) is the second lowest within this INOSS-group (47.2%).

Furthermore, Belgium has the second lowest peripartum hysterectomy rate (3.1 (95% CI 2.6-4.0) per 10,000 deliveries) in an international comparison study between ten INOSS countries (range 3.0 to 7.8 per 10,000 deliveries) (unpublished data). And the prevalence of eclampsia applying the UKOSS criteria (Schaap et al., 2014) is 1.6 (95% CI 1.2-2.1) per 10,000 deliveries in Belgium, which is very low compared to the prevalence in the UK (2.7 (95% CI 2.4-3.2) per 10,000 deliveries) (Knight, 2007) and the Netherlands (5.4 (95% CI 4.6-6.2) per 10,000 deliveries) (Schaap et al., 2014).

### Recommendations based on B.OSS results

The B.OSS findings give rise to practical recommendations: which changes should be made in clinical practice to improve the quality and safety of obstetric care?

Based on the study of uterine rupture, (Vandenberghe et al., 2016) we believe that obstetric care in Belgium would benefit from a nationally adopted guideline on trial of labour after caesarean section (TOLAC). This guideline should result from the comparison and fusion of the existing international guidelines (SOGC, 2005; Sentilhes et al., 2013; RCOG, 2015; ACOG, 2017) and have the consent of the professional associations for OB-GYNs, the professional associations for midwives and the College of Physicians for Mother and Newborn. The guideline should be developed to promote safe TOLAC in appropriate candidates on one hand, in order to enhance the low current TOLAC rate in Belgium. On the other hand, the guideline should prevent unsafe procedures, which have been observed among the uterine rupture cases in the B.OSS study. Furthermore, the guideline should include recommendations on the mode of delivery in women with other uterine surgical procedures (such as myomectomy, hysteroscopic resection of a uterine septum or salpingectomy), in whom a management similar to women with previous caesarean section is justified. A follow-up registration of uterine rupture by B.OSS should be planned over time, to monitor the effect of and compliance with the national guideline.

Based on the study of peripartum hysterectomy and arterial embolisation, (Vandenberghe et al., 2017) we believe that the management of obstetric haemorrhage in Belgium could be further improved if all women with abnormal placentation were managed in a tertiary referral centre. Therefore, we need to enhance the awareness of risk factors for abnormal invasive placenta (AIP) on the part of clinicians in order to improve the identification of women at risk and increase the antenatal transfer to tertiary referral centres in case of suspicion. This can be achieved by, besides the development of a new national guideline, the propagation of the B.OSS publication accompanied by clear instructions and ultrasound criteria for AIP, through the websites of B.OSS and the professional associations for OB-GYNs and through presentations on regional and national conferences. More influential would be a restructuring of (obstetric) health care in Belgium by the government, making referral to tertiary referral centres obviously and compulsory in case of certain conditions, such as AIP. A follow-up registration by B.OSS should include all women with massive obstetric haemorrhage, using the INOSS international consensus definition,(Schaap et al., 2017) and all women successfully managed with other second-line measures (such as intra- uterine balloon tamponade), to get the overall picture of postpartum haemorrhage in Belgium. Furthermore, a case-by-case in-depth analysis would be interesting to reveal whether the hysterectomies and arterial embolisations performed in this study were appropriate or preventable. Based on the current findings, we hypothesise that hysterectomies may be performed more easily in smaller maternity units, while there may be an overuse of arterial embolisations when this service is easily accessible, without first resorting to second-line measures. Based on the study of eclampsia, we believe that there is scope for improvement by increasing the awareness of health care providers, mainly general practitioners and emergency room doctors besides OB-GYNs and midwives, of the use of magnesium sulphate as the first choice treatment in case of an eclamptic fit. In current B.OSS study magnesium sulphate was administered as first treatment in only 54% of the women. This calls for another national guideline.

## Discussion: How can we further improve the Belgian Obstetric Surveillance System?

### Strengths and limitations

Although relying on the willingness of busy clinicians, B.OSS achieves an excellent participation rate and response rate. The success of B.OSS demonstrates the hunger for information on severe maternal morbidity on a national basis. Rates gradually dropped in the latest years however, blamed partly on the extremely rare conditions currently under investigation. To further motivate clinicians and boost the response rate, we suggest several action points. Firstly, starting new studies that investigate more common conditions that are relevant for every obstetrician. Secondly, proclaiming the results of the first B.OSS studies in presentations and publications and proclaiming the impact on quality of care, when recommendations have been implemented over time. Thirdly, rewarding the maternity units that register in a pay- for-quality project or making the B.OSS registration mandatory by the government.

Thanks to its methodology, that is the prospective collection of cases through an active search on a monthly basis according to the principle of nothing-to-report, the number of missed cases (false negatives) can be reduced to a great extent. Besides, the number of false positives is limited to a minimum thanks to the detailed data collection forms that confirm the diagnosis. However, there is currently no appropriate way to check the data of B.OSS for completeness and correctness. The validity of B.OSS data could be further improved by involving more than one clinician per maternity unit, such as a senior midwife, an obstetric anaesthesist or an intensive care specialist. This would follow the lead of the UKOSS, where four clinicians are nominated in each hospital (Knight et al., 2005).

The launch of the B.OSS website (http://www.b-oss.be) substantially reduced the workload of the research team and contact persons by enabling web- based data collection. For example, the monthly reporting is now easily done in three clicks by an email link. However, the commitment to the B.OSS project does involve additional work to contact persons on top of their clinical practice. Moreover, clinicians are regularly approached to participate in all kind of surveys. To further improve the workload, attention must be paid to keep data collection forms of future surveys more concise and clear. Furthermore, future data collections will adhere to the INOSS Core Dataset (ICoD), a standardized minimum dataset developed by the International Network of Obstetric Survey System, to enhance the comparison of data between different countries. Over time, it would be most expedient if B.OSS could join the federal project Healthdata.be (WIV-ISP), a platform developed to reduce the administrative burden of clinicians. The Healthdata-platform aims to standardise and harmonise the registration and storage of health data, while absolute confidentiality is guaranteed. It enables to gather health data in a standardised way from different health care institutions and health care providers using many different medical record systems, by implementing a specifically developed software program. Since start in 2014, they succeeded to successfully integrate 42 registries into the platform. We await further development and results of Healthdata, to assess whether the B.OSS data collection could be (partially) covered by this platform.

### Addition of audit studies

The B.OSS surveys give an overall picture of the severe obstetric complications under surveillance, by an epidemiological description of prevalence, risk factors, management and outcome for mother and child. They do not allow to make firm conclusions on the quality of care provided to individual women included in the surveys. This requires a case-by- case in-depth analysis based on the full medical records, thereby assessing the management against relevant guidelines in order to make a judgement whether there is evidence of quality of care concerns. A structured review of near-miss cases by a multidisciplinary team of appointed experts is done on an annual basis in the UK since 2014, in a programme additional to the Confidential Enquiries into Maternal Deaths (CEMD) and the UKOSS, the so-called Confidential Enquiries into Maternal Morbidities (CEMM) (Knight et al., 2014). To set up audit studies in addition to B.OSS surveys, we first need to develop the practice of performing confidential enquiries in Belgium, and preferably have drafted nationally adopted guidelines against which to assess the quality of care.

### Maternal mortality

Belgium can no longer lag behind its neighbouring high-income countries and other even low-and middle-income countries which have a well- established Confidential Enquiries into Maternal Deaths (CEMD) since many years. Now that B.OSS has a firm position, supported by the great majority of Belgian OB-GYNs, it is the right time to add maternal mortality to the project. There is clearly a need for a structured maternal mortality register in Belgium, since the B.OSS research team was asked by several clinicians where to report a case of maternal mortality. The added-value of a CEMD is obviously demonstrated by countries as UK and the Netherlands, where still half of cases is shown to be preventable and due to issues related to quality of care. The College of Physicians for Mother and Newborn is currently taking the lead and appointed a working group to organise a structured Belgian CEMD.

## Conclusion

The Belgian Obstetric Surveillance System has become a successful institute to monitor severe maternal morbidity in Belgium. The excellent participation rate and response rate demonstrates the willingness of Belgian OB-GYNs and their hunger for information on obstetric complications on a national basis. The results of the first studies suggest the need to develop nationally adopted guidelines. Furthermore, the results invite to critically evaluate the current organisation of obstetric health care in Belgium. B.OSS aims to monitor the impact on patient safety in future surveys, when guidelines and recommendations are put into practice.
